# Anticancer Conjugates and Cocktails Based on Methotrexate and Nucleoside Synergism

**DOI:** 10.4137/cmo.s2113

**Published:** 2009-03-20

**Authors:** Anthony R. Vortherms, Hester N. Dang, Robert P. Doyle

**Affiliations:** Department of Chemistry, Syracuse University, Syracuse, NY 13244-4100, U.S.A.

**Keywords:** nucleosides, folic acid, cocktail therapy, conjugates, PEG

## Abstract

Conjugates of methotrexate (MTX) and the nucleoside analogs 3-azidodeoxythymidine (AZT), iododeoxyuridine (IUdR) and dideoxycytidine (ddC) linked using poly(ethyleneglycol) are presented. In vitro cytotoxicity assays of the conjugates against drug resistant ovarian cell line A2780/AD are preformed and comparisons made to such assays performed for unconjugated (cocktail) systems. All systems tested were inactive, or had low activity, at 24 h. After 72 hr incubation however, the cocktails of MTX and AZT, IUdR or ddC showed high cytotoxicity in the low nanomolar range. The conjugates were only very moderately active with IC_50_ values in the [0.1 to 1.0 mM] range. Conjugation of the antifolate to the nucleoside analogs has it seems reduced the activity significantly when compared to a cocktail of the components, indicating a conjugate approach is unlikely to translate into success in vivo. The positive note comes from the observation that by combining two of the new conjugates, namely those based on MTX with IUdR or AZT, an IC50 at 24 hours of ~ [180 μM] was produced.

## Introduction

Targeted drug delivery is a rapidly growing field of chemotherapeutics. The goal of targeted drug delivery is to minimize the loss of healthy tissue that occurs during traditional, systemic chemotherapy. The ability to selectively target tumours greatly improves a drug’s therapeutic index and lowers the treatment burden on the patient. To achieve this goal, chemotherapeutics are designed to have two (or more) major components. One such component is the cytotoxic compound, with the other being the targeting agent. This latter moiety has driven the search for agents that will deliver the cytotoxic component to unique markers of tumours such as prostate specific membrane antigen or over-expressed folate receptor (FR).^1^ Indeed, the FR has proven highly successful in targeting studies, benefiting from an overexpression in certain tumor lines and the high binding affinity of FR (K_D_ ~ 0.42 × 10^9^ M) for folate.^2^

**Figure 1.**
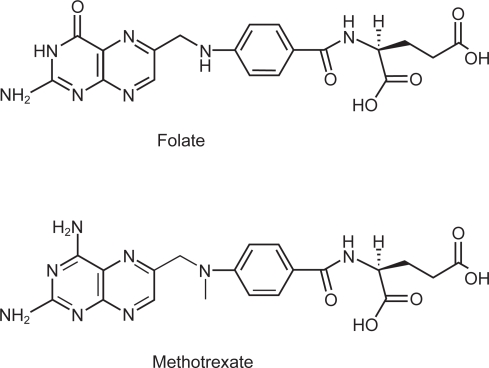
Folic acid (vitamin B_9_) and the antifolate, methotrexate (MTX). Note the methylation of the amine of the *p*-amino benzoic acid moiety and amination of the pterin carbonyl on conversion from folate to MTX.

The use of antifolates in cancer therapy has also received considerable attention over several decades because of their ability to inhibit folate dependant enzymes. These enzymes are of particular interest in cancer chemotherapeutics because they are vital for DNA synthesis. Methotrexate (MTX) (see Fig. 1), the most widely used antifolate, interferes with *de novo* thymine synthesis for example.^3^ The primary target of MTX is dihydrofolate reductase (DHFR),^4^ which is responsible for reducing dihydrogenfolate (DHF) to tetrahydrofolate (THF). MTX has also been shown to be an inhibitor of thymidylate synthase^5^ (TS), which catalyses the methylation of uracil to thymine. Antifolates, because of their structural similarities with folate, can take advantage of the same delivery pathways as for folate in vivo. There is an important *caveat* for FR however and it is the fact that the FR has an approximately 100-fold lower affinity for MTX over folate.^6^ Using MTX as a successful targeting moiety for the FR then is unlikely.

Delivery of MTX through the reduced folate carrier (RFC) however, coupled with its demonstrated anticancer properties, still make this an intriguing system for incorporation into new bioconjugates. The RFC has a high affinity for MTX with a K_m_ of 5–10 μM (Folate; K_m_ = 200–400 μM).^7^ Using this mode of cellular entry and combining MTX with systems that contrast, but complement, its intracellular activity (e.g. interfering with DNA synthesis/replication) may provide new systems whose synergism provides activity greater than the sum of its parts.

**Figure 2.**
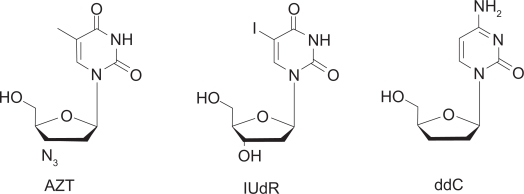
Nucleoside analogs 3-azidodeoxythymidin, 5-Iodouracil, and 2’ 3’ dideoxycytosine.

The complementary system we decided to investigate, in combination with MTX, was that of nucleoside analogs. We chose nucleosides because of their potency *in vivo.* Both MTX and nucleosides analogs suffer from general systemic delivery and a lack of more specific targeting (relative to the FR). This usually requires greater doses be administered (when feasible and appropriate) to acquire the necessary cytotoxic concentration. This often makes these compounds unsuitable for use on their own. Nucleoside analogs have been primarily examined for their ability to inhibit reverse transcriptase, with 3-azidodeoxythymidine (AZT) (Fig. 2) being one of the most notable examples.^8^ Nucleoside analogs, as with antifolates, also have the ability to inhibit enzymes necessary for *de novo* thymidine synthesis, such as thymidylate synthase (TS) and thymidylate kinase (TK). Alternatively they can be incorporated into a growing strand of DNA, and either cause strand termination, or produce a kink in the DNA that prevents replication.^9^

We were further inspired to conjugate these systems by reports that combinations of MTX and 5-flurouracil^10^ or AZT^11^ overcame resistance to MTX in certain cell lines. Our aim for this work then was to compare and contrast, and investigate possible synergy in, MTX and nucleoside cocktail and conjugate systems.

**Figure 3.**
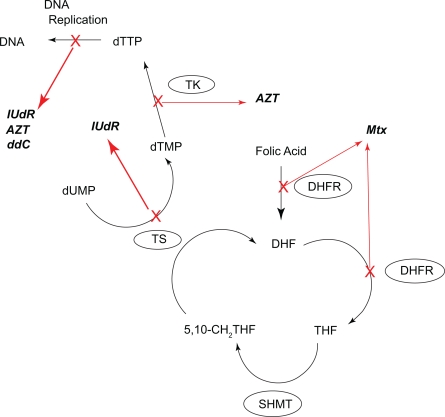
Thymidylate synthesis pathway and the enzymes targeted by methotrexate and nucleoside analogs. Enzymes are show in circles and X’s indicate inhibition of that process bv the drug (shown in italics).

The hypothesis was that the conjugated systems would provic greater toxicity, especially at earlier time points, than the simple mixed systems. Both the mixed and conjugated systems should provide some degree of synergistic enzyme inhibition (see Fig. 3) but the conjugated systems should allow for greater uptake, presumable through the reduced folate carrier via MTX, thus ensuring the nuceloside analog is also delivered. In additon to acting as a spacer, the linker unit chosen, namely polyethyleneglycol (PEG), may prevent cellular efflux of the conjugate after uptake, again providing for greater toxicity for the conjugates over the cocktails, by increasing the residencey time of both the MTX and conjugated nuceloside analog. This work focused MTX with three nucleoside analogs, namely AZT, iododeoxyuridine (IUdR) and dideoxycytidine (ddC) (see Fig. 2).

## Results and Discussion

**Figure 4.**
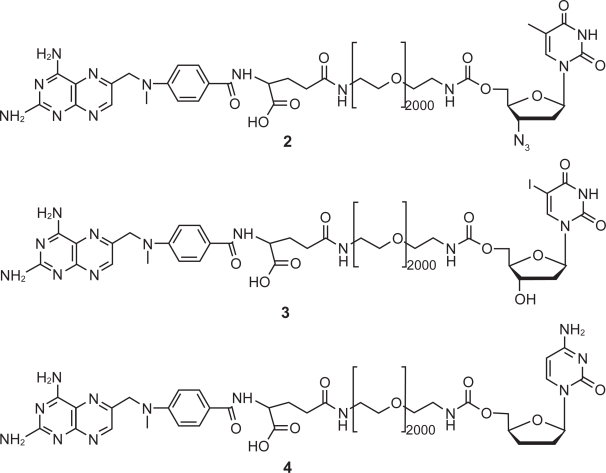
Nucleoside conjugates of γ-MTX-PEG (**1**) with AZT (**2**), IUdR (**3**)**,** and ddC (**4**).

All of the conjugates produced used classic, facile ‘click’ chemistry. It is necessary, upon conjugation, to separate out the two isomers produced. There is an active γ-isomer and an inactive α-isomer produced by tandem coupling through the α-, and γ-carboxylic acids of FA or MTX. The MTX-PEG building block (**1**) was synthesized using a similar approach to that used by us for the preparation of Folate-PEG-NH_2_.^12^ The desired γ-isomer was then purified by ion exchange chromatography using an ANX column. The drugs were synthesized using the same procedure for each of the three nucleoside analogs. Briefly, MTX was activated with dicyclohexylcarbodiimide (DCC) and N-hydroxysuccinimde (NHS). The activated MTX was added to a solution of PEG, and then purified. Previous work on the conjugation of MTX to PEG show a racemization of the MTX.^14^ The enantiomers were not purified out and were reacted together in the subsequent conjugation. The nucleoside analogs were directly linked to (**γ−1**) by CDT coupling to create the three conjugates studied (see Fig. 4).

Over 24 hours, the conjugated drugs show no cytotoxicity over the concentrations tested (up to [1 mM]). Combining MTX with AZT or IUdR as a cocktail however gave IC_50_ values at ~[6 mM] at 24 hours, a significant improvement on that noted for conjugates of the same components. Interestingly, when MTX was combined with ddC, the cocktail did not produce an observed IC_50_ value at 24 hours, as noted for MTX/AZT and MTX/IUdR. Unlike AZT and IUdR, ddC does not inhibit one of the primary enzymes of the thymidine synthesis pathway, a fact that might explain the lack of synergism.

Both the cocktails and conjugates of MTX with all three nucleoside analogs did however show IC_50_ values at 72 hours. At 72 hours the conjugates show cytotoxicity in the ~ [0.20–0.9 mM] concentration range. In stark contrast, the cocktails greatly affected cell viability, with IC_50_ values now in the low [nM] range.

It is clear that the combinations, rather than the conjugations, are far more effective at reducing cell viability.

Based on our observed results, we decided to see if a cocktail of the two most active conjugates, namely (**2**) and (**3**), could work in tandem to achieve cytotoxicity more akin to the free, mixed components. Such a cocktail would be expected to have the MTX uptake route, but also increased activity because of its putative ability to inhibit three of the major enzymes in thymidine synthesis, namely DHFR, TS, and TK. The cocktail of (**2**) and (**3**) shows [uM] activity over 72 hours similar to the results observed with each conjugate individually and so again far inferior to the combinations of MTX with AZT or MTX with IUdR alone, as recorded in Table 1. Importantly, however, over 24 hours the new cocktail of (**2**) and (**3**) achieved more significant toxicity, ~[0.18 mM]. This is far superior to the free components at the same 24 hour time point. The individual conjugates (**2**) and (**3**) did not show toxicity up to [1 mM] over the same time frame.

**Figure 5.**
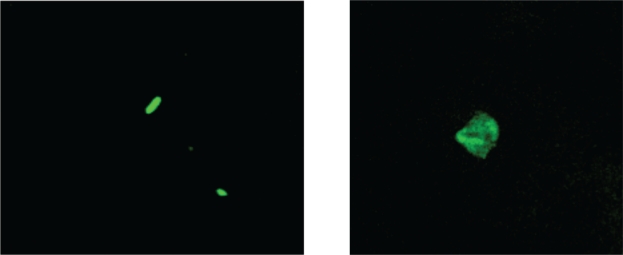
Drug uptake monitored by confocal μ microscopy in ovarian cell line A2780/AD (10X, 100X).

To confirm uptake of the conjugates, a fluorescent analog was synthesized to follow uptake via confocal microscopy.^13^ After a one hour incubation with the fluorescent system, the A2780/AD cells showed uptake as can be seen in Figure 5. Depth scanning at 1 μM per layer confirmed the compound was localized throughout the cell.

To glean further insight into the new conjugates, (**2**), (**3**) and (**4**) were also screened for DHFR inhibition. The compounds exhibited IC_50_ values in the micromolar range [~ 105–400 μM] (see Table 2). The compounds all displayed similar inhibition, which suggests that the nucleoside analog itself does not significantly contribute to the inhibition of DHFR. The results are consistent with DHFR inhibition studies reported previously with pegylated MTX systems and as with that study the conjugates were not as effective as ‘free’ MTX alone.

To explain the *in vitro* results, the stability of the compounds was examined. (**3**) was dissolved in phosphate buffered saline at a pH of 7.4, and incubated at 37 °C. Time points were taken at 24 and 72 hours. Over 24 hours, there was no sign of free nucleoside. After 72 hours, a peak consistent with free nucleoside (confirmed by 1H NMR) appeared indicating partial decomposition. While not completely stable over the time tested, the stability of the carbamate linkage could be preventing the nucleoside from being released in the cell over the earlier time points. This is consistent with previous nucleoside conjugate work reported and the observation that (**2**) and (**3**) have a greater toxicity at 72 hours than the carrier system (**1**). The delay in the release could explain the discrepancies between the conjugated and the unconjugated systems.

## Conclusion

Looking at three nucleoside analogs that have different modes of activity, we have created a series of conjugates and compared them to the activity of their ‘free’ constituents. All compositions tested were inactive, or had low activity, after 24 h. After 72 h incubation time, the non-conjugated mixtures of MTX and AZT, IUdR or ddC show a very high cytotoxic potential in the low nanomolar range, while the conjugates are only very moderately active with IC_50_ values from [0.1 to 1 mM]. Conjugation of the antifolate to the nucleoside analogues has then reduce the activity significantly when compared to a cocktail of the components, indicating a conjugate approach is unlikely to translate into success *in vivo.* The conjugation does lower MTX’s ability to inhibit DHFR in the cell free assay. However, the PEG conjugate (**1**) has a greater activity *in vitro*, so it appears that conjugating MTX is not the the major cause of the decrease in activity. Another potential cause then might be found by focusing instead on the nucleoside. Conjugation of the 5’-hydroxy group, necessary for activity in the body, would likely decrease affinity for target enzymes (TK for instance). Earlier work with a folate-nucleoside (AZT) conjugate system however showed *increased* activity over unconjugated nucleoside *in vitro.*^12^ A reason for the lack of synergism then might be the physical conjugation itself. Being connected, it may prevent the two drugs from inhibiting both of the target enzymes at the same time, requiring significantly higher doses to achieve this result. It is unlikely that the conjugates are not escaping the lysosome given the distribution observed in the confocal microscopy studies repported herein.

The positive note from this work comes from the observation that by combining two of the new conjugates produced, namely (**2**) and (**3**), an IC_50_ at 24 hours of ~[180 μM] was observed. Given this value is better than that noted even for the free componants at this same time point, this is a significant step in the right direction for these systems and one we are currently exploring further.

## Experimental Section

### Chemicals and equipment

3’-Azido-3’-deoxythymidine (AZT) was purchased from Toronto Research Chemicals, Toronto, Canada. Methotrexate (MTX) was purchased from Axis Chemicals. 5-Iodo2’-deoxyuridine (IUdR), 2’,3’-Dideoxycytidine (ddC), Polyetheylene glycol (PEG), Dicyclohexylcarbodiimide (DCC), N-hydroxysuccinimde (NHS), l,l’-Carbonyl-di-(l,2,4-triazol) (CDT), carbonylditetrazole and sodium phosphate were purchased from Sigma as the highest purity available. Chromatography grade DMSO (Sigma) was dried through a column of molecular sieves (4 A, Sigma) under dry nitrogen. Water was distilled and deionised to 18.6 MW using a Barnstead Diamond RO Reverse Osmosis machine coupled to a Barnstead Nano Diamond ultrapurification machine. An Agilent 1100 HPLC with manual injection and automated fraction collector was fitted with a Zorbax C18 analytical column (4.6 (i.d) × 30 mm) to follow reactions and a semi-prep (10 (i.d) × 200 mm) column for purification. Nuclear magnetic resonance was carried out on a Bruker 300 MHz machine. Solvent suppression and exponential transformation typically gave optimum results for NMR spectra containing Polyethyleneglycol. Electrospray mass spectrometry was performed on a Shimadzu LCMS-2010A system at a cone voltage of 5 kV. RPMI media (folate free) was purchased from the American type culture collection (ATCC). PeniStrep antibiotic cocktail and Cell stripper were purchased from Sigma Aldrich. A Thermo Multiscan EX 96-well plate reader was used at a wavelength of 450 nm. Adriamycin resistant ovarian cell line A2780/AD was a generous gift from the Fox chase cancer centre, Philadelphia. Human blood serum was purchased from SIGMA.

### HPLC purification

**(2), (3), (4)** and **(5)** were purified by reverse phase gradient HPLC on a Zorbax semi-prep C18 column (9.4 × 250 mm; Agilent Technologies) with initially 100% water run up to 10% acetonitrile over 5 minutes then up to 50% acetonitrile over 12 minutes then held for 5 minutes with a flow rate of 2 ml/min.

### Synthesis of γ-MTX-PEG-NH_2_ (1)

Methotrexate (0.392 g, 0.8 mmol), DCC (0.196 g, 0.9 mmol) and NHS (0.112 g, 0.9 mmol) were dissolved in 3 ml of DMSO and stirred overnight in a dark room. The white precipitate (reaction by-product of dicyclohexyl urea) formed was removed by vacuum filtration. Bis-Amine PEG 2000 (200 mg, 0.1 mmol) was dissolved in 2 ml of dry DMSO. The activated MTX was added drop wise and stirred for 6 hours under. (**1**) was purified by LPLC as described previously. Briefly, (**1**) was precipitated out with a hexane/acetone mixture, and then redissolved in water to give a 20 mg/ml concentration. Using a 20 ml ANX column (GE) the reaction was loaded with pure water at a flow rate of 0.4 ml/min. After the first peak was eluted the column was washed with 10% 0.1 M ammonium until the first major peak was eluted, and then increased the concentration of ammonium acetate to 60% until the desired peak eluted. The yield was 30% of the desired isomer. 1H NMR (D_2_O): δ 8.32 (s, 1H, 7H), 7.82 (d, 2H, 2’, 6’ H) 7.05 (d, 2H, 3’H, 5’H), CH 3.3–3.8 (PEG) 2.92 (m, 2H). MALDITOF MS m/z Expected 2436, and found centred at 2349 [M + H]+.

### Synthesis of γ-MTX-PEG-AZT (2)

AZT (16 mg, 0.05 mmol) and CDT (10 mg, 0.06 mmol) were dissolved in 1 ml of DMSO and stirred for 4 hours at room temperature. (1) (20 mg, 0.008 mmol) was dissolved in 200 ml of DMSO and added to the solution. The reaction was then stirred overnight at room temperature under nitrogen. The solvent was removed *in vacua* and the remaining yellow solid was re-dissolved in water. (2) was isolated by HPLC with a retention time of 14.3 min. Yield is 40% based on 1. 1H NMR (D_2_O): δ 8.62 (s, 1H, 6 H), 8.32 (s, 1H, 7H), 7.82 (d, 2H, 2’, 6’ H) 7.05 (d, 2H, 3’H, 5’H), 5.99 (t, 1H, 1’ CH) 4.48 (m, 2H), 4.47 (m, 1H) 3.5–3.8 (PEG), 2.92 (m, 2H) 2.33 (b, 4H), 1.33 (s, 3H). MALDI-TOF MS m/z Expected 2736 and found centred at 2718 [M + H]+.

### γ-MTX-PEG-IUdR (3)

^1^H NMR (D_2_O): δ 8.65 (s, 1H, 6-H), 8.32 (s, 1H, 7-H), 7.72 (d, 2H, 2’, 6’-H) 6.91 (d, 2H, 3’, 5’-H), 6.2 (t, 1H, 1’ -H) 4.48 (m, 2H), 4.47 (m, 1H) 3.5–3.8 (PEG), 2.92 (m, 2H) 2.33 (m, 4H) MALDI-TOF MS m/z Expected 2813 and found centred at 2800 [M + H]+.

### γ-MTX-PEG-ddC (4)

^1^H NMR (D_2_O): δ 8.32 (s, 1H, 7-H), 7.82 (m, 3H, 2’, 6’-H, 6-H) 7.22 (d, 1H), 7.05 (d, 2H, 3’, 5’-H), 5.99 (t, 1H, 1’ CH, 7.64 (m, 3H), 5.90 (t, 1) 4.48 (m, 2H), 4.47 (m, 1H) 3.5–3.8 (PEG), 2.92 (m, 2H) 2.33 (s, 3H). MALDI-TOF MS m/z MALDI-TOF MS m/z Expected 2675 and found centred at 2651 [M + H]+.

### Synthesis of γ-MTX-PEG-ReBQAV (5)

Re(CO)_3_-BQAV (10 mg, 0.0152 mmol), prepared by previous literature protocol^13^, was activated with CDT (3 mg, 0.0182 mmol) in DMSO for 1 hour at 37 °C. The reaction was allowed to cool to room temperature and compound (1) (20 mg, 0.0082 mmol) was added, and allowed to react overnight. The product was precipitated out with acetone/hexane mixture then purified with HPLC conditions above with a retention time of 16.8 minutes. ^1^H NMR (300 MHz, D_2_O): 8 = 8.62 (s, 1H), 8.43 (d, 2H), 8.02 (d, 2H), 7.98 (d, 2H), 7.82 (m, 4H), 7.64 (m, 4H), 7.53 (d, 2H), 3.5–3.8 (PEG) 2.48 (t, 2H), 2.92 (m, 2H), 2.06 (br, 2H), 1.77 (m, 2H) MALDI-TOF MS m/z MALDI-TOF MS m/z Expected 3122 and found centred at 3050 [M + H]+.

### *In vitro*, cell free DHFR inhibition assay

The DHFR calorimetric assay was purchased from Sigma Aldrich, and was performed according to instructions. Briefly, DHFR was mixed with inhibitor and NADPH.

Dihydrofolate was then added to the mixture and the absorbance at 330 nm was monitored over 2.5 minutes. The average slope was compared to the average slope of a control that did not have any inhibitor present. The percent of control was plotted, and a fit using a power regression line used.

### *In vitro* cytotoxicity (WSK-8) assays

The proliferation of exponential phase cultures of A2780/AD cells was assessed by colorimetric assay (WSK-8). In brief, cells were plated onto a 96 well microtiter plates at 5,000 cells per well. After a 24 hour incubation period to facilitate adherence, the RPMI media was removed and replaced with 100 or 200 μL of fresh media containing the different concentrations of drug. The cells were then incubated for 1 or 3 days at 37 °C at 95% humidity and 5% CO_2_. The media was then removed, and replaced with 100 μL fresh media containing 10% WSK-8 dye. The plates were incubated for 1.5 hours and subsequently measured at 450 nm with a 96-well plate reader. A non-linear curve fitted to the concentrations tested was used and the data plotted using Origin software. Each concentration was run in triplicate, and drug was tested 3 times. MTX has a greater affinity (100 times) for the reduced folate carrier (RFC) over folate, and has a 100 fold less affinity for the FR than oxidized folate.^11^ To fully investigate the uptake and cytotoxicity of the conjugates produced, IC_50_ values were obtained in folate deficient media.

### Confocal microscopy experiments

The A2780/AD ovarian cancer cells (150,000 cells/dish) were plated on 356 × l00 mm vented dishes. The cells were incubated at 37 °C overnight in FA-free RPMI 1640 media. To each plate was added [10 mm] of (5) (1 mL volume) the plate then incubated for 1 hour. The drug was then removed, and the cells were washed with 50 mm phosphate buffered saline (PBS; 3 × l mL). The cells were then washed with acidified saline solution (3 × l mL; 3.4 mm NaCl, pH 3.0) and fixed with ice-cold MeOH.

### Stability study

**(**3**)** was dissolved in PBS buffer 1 at pH 7.4 in a concentration of [l mM]. The solution was incubated at 37 °C for 24 and 72 hours. Samples from the solution were examined using the HPLC Eclipse XDB C18 analytical column (4.6 × 159 mm; Agilent Technologies). The method conditions were the same as the purification with a flow rate of 0.7 ml/min.

## Figures and Tables

**Table 1.** *In vitro* IC_50_ [mM] values against the A2780/AD overian cancer cell line.

**Drug**	**24 hours**	**72 hours**
MTX	>10	7 ± .9
AZT	>10	8 ± 1
MTX + AZT	6 ± 1	<1 × 10^−6^
MTX + IUdR	6 ± 1	<1 × 10^−6^
MTX + ddC	>10	<1 × 10^−6^
1	>1	0.700 ± .1
2	>1	0.43 ± .03
3	>1	0.211 ± .1
4	>1	0.960 ± .080
2 + 3	0.184 ± .072	0.101 ± .014

**Table 2.** Inhibition of dihydrofolate reductase.

**Compound**	**IC_50_ [μ, M]**
1	200
2	152
3	394
4	178
MTX	1.0
